# Influence of Language and Physician–Patient Racial Concordance on Diabetes Control: An Analysis of Consultations in California Clinics

**DOI:** 10.1007/s40615-025-02581-9

**Published:** 2025-08-15

**Authors:** Miguel Ángel Fernández-Ortega, Lilia V. Castro-Porras, Gustavo A. Olaiz-Fernández, Elizabeth Gómez-Cabrera, Jesús Alonso Martínez-Ortiz, Andrea Fernanda Aguirre-Vázquez, Alejandra Chavez-Ciriaco, Félix J. Vicuña de Anda, Elena G. Gómez-Peña

**Affiliations:** Centro de Investigación en Políticas, Población y Salud, Facultad de Medicina, Universidad Nacional Autónoma de México, Universidad Nacional Autónoma de México, 12 Ciudad Universitaria, Edificio CIPPS-Sótano y Piso 2, Cto. Centro Cultural S/N, Coyoacán, 04510 Mexico City Mexico

**Keywords:** Diabetes mellitus, Hispanics, Migrants, Language, Communication barriers, Health services

## Abstract

**Objective:**

To compare the proportion of patients with diabetes under metabolic control in three health clinics attended by Mexican doctors vs. non-Mexican doctors.

**Methods:**

Longitudinal analytical study of three clinics in California in which data from 8979 consultations in 2023 were analyzed. Control of patients with diabetes was evaluated based on the nationality of the physician (Mexican/non-Mexican), the type of consultation (first-time or follow-up), and referrals to the Nephrology department. Frequency analysis and chi-square were performed to estimate the risk of the selected outcomes (uncontrolled diabetes or referral to the nephrology service), and the Hosmer and Lemeshow goodness-of-fit test was applied.

**Results:**

Better control was observed in consultations of patients with diabetes seen by Mexican physicians compared to non-Mexican physicians OR = 1.62 [1.42, 1.84]. Likewise, the percentage of patients referred to Nephrology was almost triple by non-Mexican physicians compared to Mexican physicians OR = 2.69 [1.84, 3.92].

**Conclusions:**

The cultural and linguistic concordance between Mexican physicians and Spanish-speaking patients may contribute to improved diabetes control and could lead to greater engagement with primary care services among newly diagnosed diabetes patients.

## Introduction

This work evaluates the impact of AB 1045/Chapter 1157 (the Licensed Physicians and Dentists from Mexico Pilot Program), enacted in the State of California, USA (2002) [1], on the medical care provided to patients with diabetes in three health clinics (22 medical units) in the counties of Monterey, San Benito, and Tulare, California. The Pilot Program includes 30 Mexican physicians from the following specialties: family medicine, internal medicine, pediatrics, gynecology, and obstetrics, who will practice for three years in California community health clinics serving a predominantly immigrant Latino population. This initiative aims to increase access and satisfaction among monolingual patients.

Immigrant status is a social determinant of health. It is not only due to the conditions in which the displacement occurs, but also in the cultural, social, political, economic, epidemiological, and genetic contexts. These conditions and contexts affect the health-disease process of the people and communities in which they settle [2, 3]. Disparities faced by immigrants in terms of language, access to health services, high-risk jobs, overcrowding, and social isolation, among others, increase morbidity and mortality rates in the Hispanic population in the United States of America (USA) [4–6].

Immigrants face several challenges from the moment they arrive in the U.S. regarding acculturation. Perhaps the most essential challenge is diet. In the host community, such a diet is characterized by high amounts of carbohydrates and saturated fats, as well as portions exceeding an individual’s daily caloric needs. This favors the development of chronic degenerative diseases. [7–9] In this regard, several authors have reported that as immigrants become acculturated or assimilated to the U.S. lifestyle, the prevalence of diseases such as diabetes, ischemic heart disease, and obesity, among others, increases [10–16]. Both the disparities and the acculturation process in immigrants result in lower quality of health care for Latino patients, mainly Mexicans [4, 17, 18].

In 2021, 14.7% of the U.S. adult population was reported to have diabetes mellitus, 11.3% were already diagnosed, and 3.4% had it but did not yet know it, mainly men over 65 years of age [19]. The immigrant population that year, 11.7% of Hispanic residents had diabetes (11.1% were Mexican), in contrast to 6.9% of non-Hispanic whites (6.9%) [20]. In addition, Latinos had twice the risk of lower limb amputation. They also had three times the risk of end-stage renal disease, which was the leading chronic complication in 2020 (39.2%) [21].

A study conducted in health clinics in four California counties in 2021 concluded that the immigrant population’s main barrier to health care access and satisfaction was language, as only 5.4% of physicians spoke Spanish in California [22, 23]. The lack of linguistic concordance highlights racial disparities and renders an adequate healthcare system for this community unaffordable [24, 25]. Physicians must understand the influence of social and cultural contexts on their patients’ health to achieve positive disease outcomes [26]. According to some studies, language and physician–patient racial concordance in monolingual Hispanic patients favor adequate adherence to treatment, good metabolic control, and limit the occurrence of complications in diseases such as diabetes [4, 27–30]. Another study on physician–patient communication and compassionate care reported that understanding how patients think and feel reduces worry, distress, pain, or suffering and decreases serious complications in patients with diabetes by 40–50% [31].

The objective of this study was to compare the proportion of consultations of patients with diabetes in metabolic control in three health clinics attended by Mexican versus non-Mexican physicians, as established by the standards of the Uniform Data System Reporting Requirements for 2023 Health Center Data. We hypothesize that ethnic concordance between Mexican physicians and patient consultations will result in better diabetes control among patients of Hispanic origin.

## Methods

This is a cross-sectional study with secondary data analysis. The sample for the analysis was drawn from pooled data provided by health authorities from twenty-two medical units of three community health clinic networks in Tulare, Monterey, and San Benito counties in Central California in 2023.

In these counties, the population using the clinic reaches up to 90% of Hispanic origin (2024), primarily comprising first- and second-generation immigrants living in rural communities, whose occupations are predominantly in fields such as agriculture, service, and domestic work, with an average monthly family income of $3147. For confidentiality, the clinics were arbitrarily identified in this study as Clinic One (C1), Clinic Two (C2), and Clinic Three (C3). The medical consultations included in this study were those obtained from the annual medical reports during 2023. Since the data were from pooled statistics, participants didn’t need to sign an informed consent form approved by an Ethics Committee and/or Research Committee. The total number of Mexican medical providers in the study was 22, and the number of non-Mexican physicians was 30 in the three clinics.

### Eligibility Criteria

All diabetic patients treated at the three clinics studied during 2023, regardless of their age, sex, or marital status, were included in the study.

### Outcome Variables

“Diabetes was considered uncontrolled if the HbA1c level was 9.0% or more.”

The payment records provided to nephrology providers for the consultation determined the referral to the nephrology service. The affirmative response resulted in the patient being categorized as a referral.

### Covariables

Grouped data on the number of consultations, the nationality of the treating physician (Mexican or non-Mexican physician), whether the consultation was a first-time or follow-up consultation, and the clinic where the patient was seen. To classify the treating physician according to their nationality, data from the clinic database were used. There is a record of each consultation in this database, including the name of the treating physician and his/her nationality. Mexicans are those trained in Mexico and hired under AB 1045. Physicians of any nationality who worked in the clinics studied, performing the same functions as Mexican physicians, were considered non-Mexicans.

### Analysis Plan

#### Descriptive Analysis

The frequency and percentage of items related to the characteristics of the consultation they received were described. The Chi-square Test of Independence was performed to determine whether there is a relationship between the physician’s nationality and the categories of the variables. Results were presented by nationality of the treating physician and for the total of each sample.

#### Bivariate Analysis

Bivariate logistic models were developed to estimate the risk of outcomes, including uncontrolled diabetes (yes/no) and referral to the nephrology service (yes/no), with the nationality of the treating physician considered as an independent variable.

#### Multivariate Analysis

To estimate the adjusted risk of the selected outcomes (uncontrolled diabetes or referral to the Nephrology department), the nationality of the treating physician was considered as exposure, and the clinic in which care was received was used as the adjustment variable. We reported the odds ratios along with their 95% confidence intervals. To validate the models, the resulting multivariate adjusted logistic model was subjected to Hosmer and Lemeshow’s goodness-of-fit test (1).

Graphs were presented by the nationality of the treating physician and the clinic of care, showing the estimated probability of being in uncontrolled diabetes and being referred to the Nephrology department, based on the adjusted logistic models.

For all analyses, statistical significance was considered when p-values were less than 0.05. Analyses were performed using the Stata software version 18.

## Results

### Descriptive Results

The total sample was 8979 consultations, comprising 2,211, 5,999, and 769 consultations for C1, C2, and C3, respectively. The total number of consultations provided by Mexican physicians was 38.1%. However, virtually all consultations in C3 were provided by Mexican physicians, while in C1, almost all consultations were provided by non-Mexican physicians (95.5%). C2 had the highest percentage of consultations, reaching 66.8%. Regarding this C2 clinic, the difference in the number of consultations provided by physicians of both nationalities was less than 15%, with 42.6% of consultations offered by Mexican physicians and 57.4% by non-Mexican physicians. In all three clinics, the majority of consultations were follow-up consultations (more than 70%), regardless of the nationality of the treating physician. In addition, more than half of the patients having follow-up consultations reported having controlled diabetes mellitus disease, with HbA1c figures below 9.0%. However, C2 showed a 12.4 percentage point difference in favor of consultations performed by Mexican physicians. Of the total consultations with patients having diabetes that this group of specialists provided, 75.6% achieved adequate disease control. Regarding the number of consultations in which patients were referred to the Nephrology department, the percentage was almost three times higher in non-Mexican physicians compared to Mexican physicians (7.1% vs. 1.6%, respectively). On the other hand, the percentage of first-time consultations is higher among Mexican physicians (27%) compared to non-Mexican physicians (20%) (Table 1).

**Table 1:** Description of consultations to patients with diabetes by clinic and by nationality of the treating physician

	**One**			**Two**			**Three**			**Total**		
**Number of consultations n (%)**	**2211 [24.6]**			**5999 [66.8]**			**769 [8.6]**			**8979 [100]**		
	**Mexican ***n* [%]	**Non-Mexican ***n* [%]		**Mexican ***n* [%]	**Non-Mexican ***n* [%]		**Mexican ***n* [%]	**Non-Mexican ***n* [%]		**Mexican ***n* [%]	**Non-Mexican ***n* [%]	
Number of consultations	100 [4.5]	2111 [95.5]		2555 [42.6]	3444 [57.4]		768 [99.9]	1 [0.1]		3423 [38.1]	5556 [61.9]	
Diagnosis time			*			*			ns			*
***Newly diagnosed***	26 [26.0]	338 [16.0]		770 [30.1]	794 [23.1]		134 [17.4]	0 [0.0]		930 [27.2]	1132 [20.4]	
***Follow-up***	74 [74.0]	1773 [84.0]		1785 [69.9]	2650 [76.9]		634 [82.6]	1 [100.0]		2493 [72.8]	4424 [79.6]	
Diabetes controlª			*			*			ns			*
***Controlled***	35 [47.3]	1264 [71.3]		1350 [75.6]	1674 [63.2]		436 [68.8]	1 [100.0]		1821 [73.0]	2939 [66.4]	
***Uncontrolled***	39 [52.7]	509 [28.7]		435 [24.4]	976 [36.8]		198 [31.2]	0 [0.0]		672 [27.0]	1485 [33.6]	
Referral to Nephrologyª			*						ns			*
***No***	71 [95.9]	1576 [88.9]		1754 [98.3]	2531 [95.5]		627 [98.9]	1 [100.0]		2452 [98.4]	4108 [92.9]	
***Yes***	3 [4.1]	197 [11.1]		31 [1.7]	119 [4.5]		7 [1.1]	0 [0.0]		41 [1.6]	316 [7.1]	

ªPatients having follow-up consultations; Chi-Square Test of Independence *p< 0.01

### Inferential Results

When estimating the odds ratio in follow-up consultations with patients having uncontrolled diabetes mellitus, there was a 62% greater probability that the treating physician was non-Mexican (OR = 1.62 [1.42, 1.84]), regardless of the clinic providing the consultation. In addition, the risk of the patient being referred to the Nephrology department was more than double when the treating physician was non-Mexican (OR = 2.69 [1.84, 3.92]). These results were statistically significant (Table 2).

**Table 2:** Odds ratio (95%CI) for uncontrolled diabetes and for referral to nephrology service in follow-up patients with diabetes

	Odds Ratio	95% CI	*p*-value
Uncontrolled diabetes			
Nationality of treating physician			
* Mexican (Reference)*	1		
* Non-Mexican*	1.62	[1.42,1.84]	<0.001
Referral to nephrology service			
Nationality of treating physician			
* Mexican (Reference)*	1		
* Non-Mexican*	2.69	[1.84, 3.92]	<0.001

### Clinic-adjusted models

**Fig. 1:**
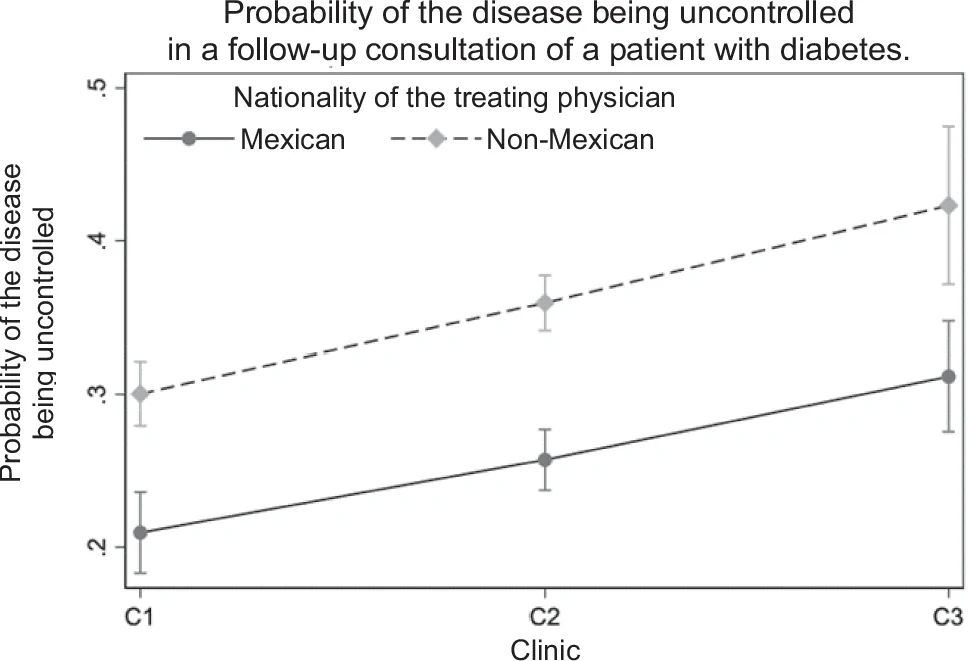
Probability of the disease being uncontrolled in a follow-up consultation of a patient with diabetes

Figure 1 shows the estimated probability of uncontrolled diabetes, stratified by the nationality of the treating physician and by clinic. The results indicate that this probability is consistently higher when the physician is non-Mexican, regardless of the clinic. This suggests a potential association between physician nationality and differences in diabetes management.

**Fig. 2:**
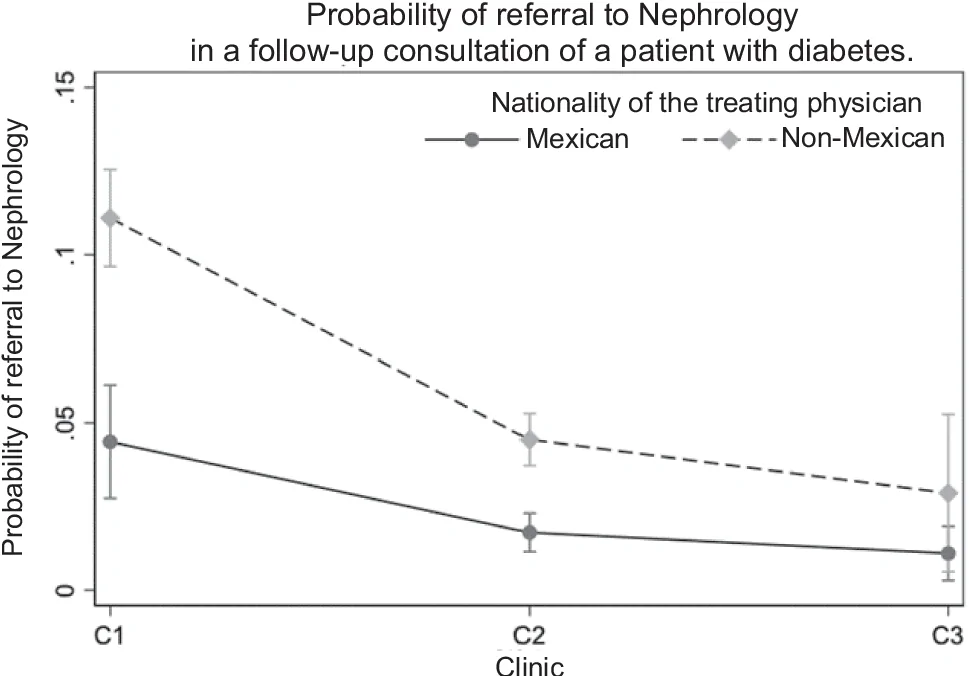
Probability of referral to Nephrology in a follow-up consultation of a patient with diabetes

Figure 2 presents the estimated probability of referral to the Nephrology department, according to the treating physician’s nationality and clinic. The models show that, in Clinics 1 and 2, patients are significantly more likely to be referred when treated by non-Mexican physicians. This finding suggests potential differences in clinical decision-making or referral practices among physicians with varying backgrounds.

## Discussion

Inequalities experienced by immigrants in the U.S. can lead to a decrease in life expectancy of up to 10 years compared to those who have formal employment, speak English [23], and have a better socioeconomic status that facilitates access to quality medical services [5, 32]. In 2021, it was reported that the sociodemographic profile of these communities studied, before the arrival of doctors from Mexico (baseline evaluation), was that 71% of the users were Latinos, housewives, farm and service workers, and 64% had basic education. In addition, they were primarily first- and second-generation immigrants. By 2023, nine out of ten patients were Latino [21, 33]. The limited access to medical care due to informal employment and the increased risk of developing a metabolic disease during the acculturation process (obesogenic diet and/or the limited time available to prepare foods of high nutritional value at home) places people with diabetes of Hispanic origin, especially first-generation immigrants, as a highly vulnerable group in terms of health [3–5, 7].

The high prevalence of diabetes in this community [18, 20] compels medical providers to perform early detection and adequate metabolic control to improve patients’ quality of life and decrease mortality rates [3]. According to the American Diabetes Association (ADA), a person is in glycemic control when blood glucose numbers are < 100 mg/dl fasting, < 140 mg/dl postprandial, or HbA1c < 5.7% [19]. However, we took the figures from the Uniform Data System Reporting Requirements for 2023 Health Center Data for this study. Accordingly, the indicator established for health clinics to report patients with poor glycemic control is HbA1c > 9.0% (Health Center Program, 2023) [34]), which all providers must follow when reporting their medical records. In this regard, determining the number of patients with good metabolic control is a challenging task. This is because many uncontrolled patients, within the 5.7% and 9.0% HbA1c ranges, were not considered in the report. In the short or medium term, it may lead them to suffer some chronic complications of this disease [6, 19].

The health inequity faced by immigrants due to the lack of cultural and linguistic competence among physicians [21, 23, 35, 36] may lead topoor adherence to treatment and the consequent evolution of the disease towards complications, such as vasculopathy, diabetic retinopathy [37], ischemic heart disease, cerebrovascular disease, and nephropathy. In this regard, nephropathy is the main complication in Latinos, and the risk of developing it and reaching terminal stages is estimated to be three times higher [3, 18, 20, 33]. The results presented in this work align with those of other studies, such as the “Northern California Diabetes Study (DISTANCE),” which have demonstrated that linguistic and ethnic concordance between doctor and patient can enhance therapeutic adherence, metabolic control, and patient participation in managing their chronic conditions [23, 38, 39].

In this study, we analyzed the referrals from internists and family physicians to the nephrology department. The number of referrals to Nephrology by non-Mexican physicians was more than twice that of Mexican doctors. This may be explained by the fact that they had treated the established patients seen by Mexican physicians for less than two years. On the other hand, non-Mexican physicians had more time for follow-up, which made the identification of chronic complications more evident. Furthermore, most consultations continue to be performed by non-Mexican physicians, since they attend to more than 60% of the consultations. The higher likelihood of follow-up visits and nephrology referrals among patients treated by non-Mexican physicians also suggests potential differences in clinical decision-making or referral practices. This may reflect a more proactive approach to managing diabetes complications, such as nephropathy, but also raises questions about continuity of care and provider-patient dynamics that merit further exploration.

In this study, consultations with Mexican physicians resulted in better diabetes control compared to consultations with non-Mexican physicians. This fact does not imply that Mexican physicians are superior to those from other countries. This may correspond to what has been reported by various authors regarding the importance of cultural, linguistic, ethnic, and racial concordance between physicians and their patients [21, 34]. Such concordance enables patients to feel more connected to their physician, to better understand therapeutic indications, to improve treatment adherence, and to resolve their concerns. Additionally, there is a greater understanding and respect for their beliefs and traditions [21–25, 27, 28, 35, 40, 41]. Similarly, these arguments may have influenced the increase in patients who came to the clinic for the first time and preferred Mexican providers. The linguistic, cultural, and racial concordance provided by the AB 1045/Chapter 1157 (the Licensed Physicians and Dentists from Mexico Pilot Program) [31] is a viable alternative to these social determinants that produce health inequity for monolingual Hispanics [21, 24, 34, 41]. The AB 1045 program arose as a legislative response to reduce health disparities through the participation of Mexican physicians in rural areas with coverage difficulties for the predominantly immigrant and monolingual population, farm workers, service workers, and homemakers. It is of a temporary nature, with three-year contracts for Mexican physicians, bilingual and with medical specialties equivalent to those of North American physicians working in those localities, serving a predominantly Mexican Hispanic population.

Some limitations of this study were the following: the HbA1c criterion used by the “Uniform Data System Reporting” for reporting patients with diabetes with poor control of the disease (indicator > 9.0%) implies that many patients with poor metabolic control (HbA1c > 5.7%) according to the ADA were not included in this report. Additionally, no clinical information was available on the patients analyzed since the information only corresponded to the characteristics of the medical consultation. Another limitation was the profile of the users of these clinics, who were predominantly Hispanic and immigrants. This fact would prevent the extrapolation of the results of this investigation to populations with different sociodemographic compositions. Additionally, the number of clinics evaluated and the number of physicians in the two clinics was relatively small; this was not proportional to the number of groups studied. In any case, it allowed for a reliable analysis. It was also important to note that the study used consultation data rather than patient data, so multiple confounding factors such as disease duration, comorbidities, treatment regimens, and adherence could not be controlled for. Lastly, it should be considered that Mexican physicians have treated patients for two years, which is insufficient time to observe the development of renal complications in each of the groups studied.

## Conclusions

Our results suggest that cultural and linguistic concordance between Mexican physicians and Hispanic patients with diabetes may positively influence metabolic control, highlighting the importance of considering cultural factors in medical care to improve health outcomes. This is a result of cultural and linguistic concordance, related to adequate doctor-patient communication in the Hispanic population. The AB 1045/Chapter 1157 (the Licensed Physicians and Dentists from Mexico Pilot Program) is a viable alternative that reduces health inequities, improves quality of care, and increases access to health services for monolingual Hispanic patients with diabetes. This legislative initiative also contributes to an increase in the number of culturally and linguistically competent providers, professionals who California universities are not currently producing to serve areas with high levels of marginalization.

Finally, this AB 1045 law is temporary, so California educational institutions must adjust their curricula to prepare bilingual and culturally competent physicians and healthcare personnel to care for and understand the Hispanic population, the largest minority in the state.

## Data Availability

The databases will be available for consultation upon request with the corresponding justification.
